# Synergistic Effects of Combined Diet and Exercise on Body Composition in Adults: A Systematic Review and Meta-Analysis

**DOI:** 10.31729/jnma.v63i292.9266

**Published:** 2025-12-31

**Authors:** Irka Dwi Fatmawati, Rahayu Sutrisno, Fajar Ari Nugroho, Mahenderan Appukutty, Yit Siew Chin, Nia Novita Wirawan

**Affiliations:** 1 Master of Nutrition Science Study Program, Faculty of Health Science, Universitas Brawijaya, Malang, East Java, Indonesia; 2 Department of Nutrition. Faculty of Health Science, Universitas Brawijaya, Malang, East Java, Indonesia; 3 Sports Science Programme Faculty of Sports Science & Recreation, Universiti Teknologi MARA, Shah Alam, Selangor, Malaysia; 4 Department of Nutrition, Faculty of Medicine and Health Sciences, Universiti Putra Malaysia, Serdang, Selangor, Malaysia; 5 Research Centre of Excellence on Nutrition and Non-communicable Diseases, Faculty of Medicine and Health Sciences, Universiti Putra Malaysia, 43400 Serdang, Selangor, Malaysia; 6 PUTRA-Strategic Health & Advanced Prevention for Excess Weight & Obesity (PUTRA-SHAPE), Faculty of Medicine and Health Sciences, Universiti Putra Malaysia, 43400 Serdang, Selangor, Malaysia

**Keywords:** *adult*, *body composition*, *diet therapy*, *exercise therapy*, *lifestyle intervention*

## Abstract

**Introduction:**

Obesity and poor body composition are major contributors to chronic disease risk inadults. This review aimed to compare the effectiveness of exercise-only, diet-only, and combined interventions in improving body composition outcomes.

**Methods:**

A systematic review and meta-analysis were conducted based on Judies published between 2016 and 2024 from Scopus, Web of Science, and ScienceDirect. Forty-six Judies involving 3,429 adults across five continents met inclusion criteria. Quality appraisal followed PRISMA and MMAT guidelines. A meta-analysis using RevMan 5.4 included six eligible Judies.

**Results:**

Combined interventions resulted in the most consistent improvements in body mass index (BMI) (SMD, -1.74; 95 % CI: -3.07 to -0.40; P = .01 ), body fat percentage (%BF) (SMD, -2.40; 95 % CI: -2.91 to -1.88; P < .00001), and total body water (TBW) (SMD, 1.54; 95 % CI: 1.04 to 2.04; P < .00001). Exercise-only and diet-only approaches also improved BMI and fat mass but had limited effects on muscle mass and TBW.

**Conclusions:**

Integrated interventions combining physical activity and dietary changes are more effective than single-modality strategies. Programs of at least 12 weeks with supervision and cultural adaptation are recommended for optimal improvements in adult body composition.

**Systematic Review Registration:**

This Systematic review is registered at PROSPERO (Reference number: CRD420251020596).

## INTRODUCTION

Obesity is the fastest-growing public health challenges of the 21st century. According to the World Health Organization (WHO), its prevalence has nearly tripled since 1975, affecting over one billion individuals worldwide.^1^ Obesity represents a preventable cause of death and is linked to cardiovascular diseases, diabetes, and certain cancers.^2-4^ Abdominal adiposity, is more strongly associated with cardiometabolic risk than total body fat and is independent predictor of coronary heart disease.^4^ Thus, interventions targeting waist circumference are crucial. Lifestyle-based strategies including physical activity and dietary changes are widely recommended.^4-6^ Yet, the comparative effectiveness of exercise-only, diet-only, and combined approaches remains inconsistent across studies.^7,8^

Existing reviews are constrained by limited geography, minimal modality-specific comparisons, and minimal stratification by intervention duration or supervision.^9^ Heterogeneity in study design, outcomes, and follow-up periods hampers synthesis and limits guidance for practice. This systematic review and meta-analysis evaluates exercise-only, diet-only, and combined interventions on key body composition to identify effective, culturally adaptable, and sustainable strategies for obesity management.

## METHODOLOGY

### SEARCH STRATEGY AND ELIGIBILITY CRITERIA

This systematic review and meta-analysis adhered to the Preferred Reporting Items for Systematic Reviews and MetaAnalyses (PRISMA) guidelines to ensure methodological transparency. The protocol was registered in the International Prospective Register of Systematic Reviews registered in March 2025 (PROSPERO; ID: CRD420251020596). A comprehensive literature search was conducted in February-March 2025 across Scopus, Web of Science, and ScienceDirect, covering studies published between January 2016 and December 2024. Databases were employed to identify additional studies. Boolean operators were applied to formulate search terms addressing nutrition and wellness programs and body composition in adult populations. Only articles published in English were included to ensure consistency in data extraction, minimize translation bias, and enhance methodological rigor, a practice commonly applied in systematic reviews.

Eligibility criteria were defined using the PICO framework. The population included adults aged >18 years, irrespective of baseline weight status, encompassing individuals with normal weight, overweight, or obesity. Interventions comprised dietary strategies (e.g., calorie restriction, intermittent fasting, Mediterranean diet), structured physical activity (e.g., aerobic exercise, resistance training, Zumba), and combined diet-exercise programs. No restrictions were applied regarding intervention duration; both short- and long-term interventions were eligible, provided they assessed at least one predefined body composition outcome. Comparators included minimal intervention or alternative strategies.

The primary outcomes were changes in body composition, including BMI, fat mass (FM), fat-free mass (FFM), body fat percentage (%BF), total body water (TBW), muscle mass (MM), waist circumference (WC), and visceral fat (VF). Studies were excluded if they targeted populations younger than 18 years, focused solely on pharmacological or surgical treatments, did not report relevant body composition outcomes, or lacked accessible full texts.

**Figure 1:**
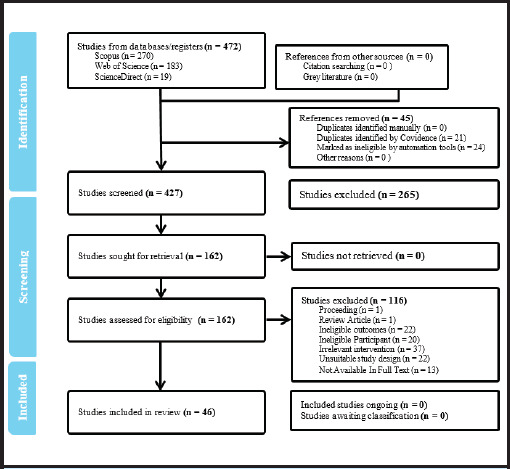
PRISMA flowchart for the study identification procedure of the meta-analysis.

The search strategy, developed using Boolean operators, identified 474 records. After removing 45 duplicates, 429 studies were screened. Following title, abstract, and full-text review against predefined criteria, 46 studies were included. Of these, 40 contributed to the qualitative synthesis and 6 to the quantitative meta-analysis (Figure 1). Screening and selection were conducted using Covidence software to ensure methodological rigor.

### DATA EXTRACTION AND QUALITY ASSESSMENT

Data extraction followed a standardized template capturing key study characteristics, interventions, and outcomes. Specific details on intervention type, frequency, intensity, and duration were extracted. Body composition indicators were prioritized, with secondary data including adherence and psychological outcomes. Quality assessment was conducted using the Mixed Methods Appraisal Tool (MMAT), suitable for evaluating heterogeneous study designs.^10^

Of the 46 studies included, 22 were randomized controlled trials (RCTs), 21 were non-randomized intervention studies, two applied mixed-methods, and one was descriptive. Quality evaluation revealed that 83 % of studies were high quality. High-quality ratings were assigned to studies with well-defined objectives, rigorous methods, and appropriate statistical analyses. The quality assessment further considered the clarity of research objectives.

### STATISTICAL ANALYSIS

Review Manager (RevMan) software version 5.4 (Cochrane Collaboration) was used for meta-analysis. Given the heterogeneity among studies, a random-effects model was adopted to estimate pooled effect sizes. Standardized Mean Differences (SMDs) with 95 % Confidence Intervals (CIs) were calculated for continuous variables. Forest plots were generated for visual interpretation. The I^2^ statistic assessed heterogeneity, with values >75 % indicating substantial inconsistency.

Subgroup analyses were pre-specified in the review protocol and performed according to intervention type (exercise-only, nutrition-only, combined), intervention duration, and study region to explore potential sources of heterogeneity. A total of six studies contributed to the quantitative synthesis. For exercise interventions, outcomes included BMI (2 studies), FM (3 studies), FFM (1 study), %BF (2 studies), and MM (3 studies). For nutrition interventions, outcomes included BMI (2 studies), %BF (2 studies), and MM (2 studies). For combined interventions, outcomes included BMI (2 studies), %BF (2 studies), MM (2 studies), and TBW (1 study).

Publication bias was not formally assessed due to the limited number of studies per outcome, though this was acknowledged as a limitation. All statistical procedures adhered to established methodological standards. However, the reliability of the conclusions depends not only on the rigor of the analyses but also on the quality, sample sizes, and consistency of the primary studies included in this review.

## RESULTS

### CHARACTERISTICS OF THE INCLUDED STUDIES

A total of 46 studies met the inclusion criteria and were synthesized in this systematic review and meta-analysis. Supplementary Table 1 presents the key characteristics of these studies, organized by continent and intervention duration. The included studies were conducted across five continents, involving a wide range of intervention modalities, durations, and delivery methods.

In America, structured interventions lasting 12 weeks or more were highly effective. For example, Gottesman et al. (2018) reported that a 26-week combined dietary and exercise intervention led to reductions in BMI (0.90) and %BF (3.10 %).^11^ Similarly, a 12-week online nutrition program by Thomson et al. (2018) achieved improvements in BMI (-0.60), FM (-2.36 kg), and WC (-0.70 cm).^12^

European studies yielded significant results with both exercise and diet-focused interventions. Hernandez-Reyes et al. (2019) implemented a 24-week physical activity intervention, resulting in BMI reduction of -10.10, FM -16.31 kg, and %BF -10.46 %.^13^ In a separate study, Rosi et al. (2020) showed that a Mediterranean diet program effectively reduced FM and %BF.^14^

In Asia, studies featured diverse interventions such as aerobic training, intermittent fasting, and virtual Zumba. Chiu et al. (2017) reported BMI reduction of -2.35 and WC reduction of -9.38 cm from a 12-week aerobic exercise.^15^ Akyilmaz et al. (2023) found that an 8-week online Zumba session reduced BMI (-3.05) and FM (-6.69 kg).^16^

By contrast, African studies showed limited effectiveness. Shandu et al. (2023) reported increases in BMI and body fat after 8-week HIIT and aerobic programs.^17^ In Australia, Horner et al. (2021) demonstrated that a 4-week supervised exercise achieved BMI reduction of -0.40 and WC reduction of -2.20 cm.^18^

### DURATION OF INTERVENTIONS

The duration of interventions strongly influenced outcomes. Short interventions (4 weeks), such as that of Horner et al. (2021), led to modest improvements in BMI, body fat, and WC, but had minimal effect on MM or TBW.^18^

Studies with 5-8-week durations (e.g., Sengün et al. (2024); Akyilmaz et al. (2023)) showed consistent improvements in BMI and FM, with some evidence of FFM increase.^6,16^ For instance, the online Zumba program by Akyilmaz et al. (2023) reduced BMI (-3.05) and FM (-6.69 kg).^16^

The 10-12-week interventions, such as those by Chiu et al. (2017) and Thomson et al. (2018), were demonstrated robust changes in all body composition parameters, including VF and WC.^12,15^ Interventions lasting 14-16 weeks added benefits related to hormonal balance and lean mass retention. Guerendiain et al. (2019) reported muscle gains alongside fat loss using a 16-week Zumba and bodyweight training regimen.^19^

The most comprehensive changes were observed in long-term interventions (≥20 weeks). Cheng et al. (2022)^20^ and Hernandez-Reyes et al. (2019) demonstrated reductions in BMI (-0.90 to -10.10) and %BF (-0.70 to -16.31).^13^ These findings indicate that while shorter interventions can rapidly reduce adiposity, longer programs are essential for holistic improvements in body composition and sustained health outcomes.

### EFFECTS OF INTERVENTIONS ON BODY COMPOSITION

Meta-analytical findings were derived from six studies included in the quantitative synthesis. Analysis of fat-free mass (FFM) from two studies showed a pooled standardized mean difference (SMD) of 0.16 (95 % CI: -0.38 to 0.71), indicating a small, statistically non-significant effect, with low heterogeneity (I^2^ = 0%).

**Figure 2:**
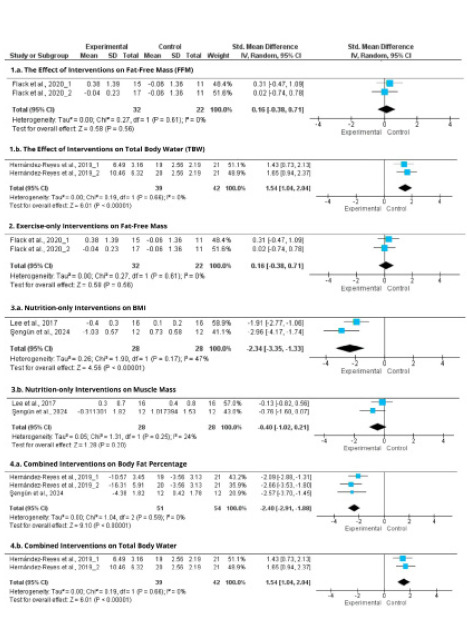
Forest plots of the effects of various interventions on body composition outcomes: (1a) Fat-Free Mass (FFM), (1b) Total Body Water (TBW), (2) FFM from exercise-only interventions, (3a) Body Mass Index (BMI) from nutrition-only interventions, (3b) Muscle Mass from nutrition-only interventions, (4a) Body Fat Percentage from combined interventions, (4b) TBW from combined interventions. Results are presented as standardized mean differences (SMD) with 95% confidence intervals (CI) using a randomeffects model. A negative SMD indicates a reduction in the outcome favoring the intervention group.

For total body water (TBW), only one study reported relevant outcomes.^13^ This study found a significant increase in TBW (SMD = 1.54, 95% CI: 1.04 to 2.04, p < .00001, I^2^ = 0%) following a combined lifestyle intervention (Figure 2). As this estimate is based on a single trial, it should be interpreted as study-level evidence rather than a pooled meta-analytic result. High heterogeneity (P = 62-87%) was observed for BMI, FM, %BF, and MM, reflecting variability across interventions, populations, and settings.

### EFFECTIVENESS BY TYPE OF INTERVENTION

**EXERCISE-BASED INTERVENTIONS**

Exercise-only interventions had mixed results. Hernandez-Reyes et al. (2019) in Europe showed large reductions in BMI (-10.10), FM (-16.31 kg), %BF (-10.46 %), and WC (-6.01 cm).^13^ Chiu et al. (2017) in Asia reported BMI decrease (-2.35), %BF (-3.97 %), and WC (-9.38 cm).^15^ Akyilmaz et al. (2023) found BMI reduction of -3.05.^16^

However, American studies showed minimal changes in BMI and FM, suggesting less impact without dietary changes.^2,7^ Shandu et al. (2023) in Africa found increased BMI and FM.^17^

Meta-analysis revealed significant reductions in BMI (SMD = -3.30, 95 % CI: -5.03 to -1.58, P = .0002, P = 62 %), and FM (SMD = -0.74, 95 % CI: -1.27 to -0.20, P = .007, P = 70 %) (Figure 2). A reduction was also observed for %BF (SMD = -0.76, 95% CI: -1.43 to -0.08, p = .03, P = 78%), although this result should be interpreted with caution given the wide confidence interval and high heterogeneity. No significant changes were found for FFM.

**NUTRITION-BASED INTERVENTIONS**

Nutrition-only interventions showed positive effects on BMI and FM. Rosi et al. (2020) in Europe reduced BMI (-3.80) and FM (-8.60 kg). Ashtary-Larky et al. (2017) reported BMI reductions between -2.00 and -2.10.^14,21^

In America, Thomson et al. (2018) demonstrated improvements BMI (-0.60), FM (-2.36 kg), %BF (-1.60 %), and WC (-0.70 cm) through online nutrition education. 12 Meta-analysis (Figures 2-3a, 3b) showed significant effects on BMI (SMD = -2.34, 95 % CI: -3.35 to -1.33, P < .00001), though %BF and MM changes were non-significant.

**Combined Interventions (Exercise and Nutrition)**

Combined interventions were most effective. Gottesman et al. (2018) reduced BMI by -0.90 and %BF by -3.10 %.^11^ Sengün et al. (2024) reported BMI reduction of -1.84 and %BF of -4.38%.^6^

Meta-analysis (Figures 2-4a, 4b) showed significant reductions in BMI (SMD = -1.74, 95 % CI: -3.07 to -0.40, P = .01, I^2^ = 87 %) and %BF (SMD = -2.40, 95 % CI: -2.91 to -1.88, P < .00001, P = 0 %). TBW improvement (SMD = 1.54, 95 % CI: 1.4 to 2.04, P < .00001, P = 0 %) was also noted, though MM increase was not statistically significant.

These findings confirm that integrative strategies addressing both diet and physical activity yield superior benefits in body composition across diverse populations and regions.

## DISCUSSION

**Duration of Intervention**

The duration of lifestyle interventions appeared to influence both the magnitude and sustainability of body composition changes, although the relationship was not strictly linear and depended on factors such as intervention intensity, supervision, and cultural context.^5,9^

Short-term interventions (≤4 weeks), such as Horner et al. (2021), reported modest reductions in adiposity but generally lacked the depth of physiological change required for lasting outcomes.^18^ Under specific conditions, however, brief high-intensity programs (e.g., Lee et al., 2017; Hu et al., 2022) achieved measurable improvements in fat mass, highlighting the role of participant engagement and training intensity.^22,23^

Interventions of 5-8 weeks demonstrated more consistent reductions in BMI, FM, and %BF, particularly in structured program.^6,16^ Nevertheless, changes in FFM and TBW were limited, suggesting this duration may be insufficient to affect lean mass or hydration without integrated support.^24^

Programs lasting 10-12 weeks were associated with more reliable improvements across outcomes, supporting evidence that meaningful remodeling of body composition—including MM and TBW—requires sustained behavioral modification.^9,12,15^ These findings align with WHO recommendations promoting ≥12-week interventions for obesity management.

Longer interventions (14-24 weeks) with professional oversight were more often linked to broader benefits in both adiposity and lean mass measures.^11,13^ Menon et al. (2022) further reported that interventions sustained over several years can reduce adiposity, improve lean tissue retention, and mitigate cardiometabolic risks.^5^

Regional variation was also evident. For example, Shandu et al. (2023) in Africa reported increases in BMI and FM during an 8-week program, suggesting that socio-cultural and economic contexts such as dietary norms, body image ideals, and resource constraints may attenuate intervention.^17,25-27^

To facilitate comparison and avoid overgeneralization, a summary table (Table 1) has been included to present outcomes by intervention duration and representative studies guidance and participant engagement.^9^

**Table 1:** Summary of Intervention Duration, Outcomes, and Representative Studies.

Duration	Key Outcomes	Representative Studies
≤4 weeks	Small, short-term reductions in BMI and WC; limited changes in FFM/MM.	Park & Nickerson, 2022^37^ (4 weeks aerobic); Horner et al., 2021^18^ (4 weeks supervised exercise)
5-8 weeks	Consistent reductions in BMI, FM, %BF; mixed effects on FFM.	Hirsch et al., 2021^38^ (8 weeks HIIT); Akyilmaz et al., 2023^16^ (8 weeks Zumba/Walking); §engun et al., 2024^6^ (8 weeks combined)
10-12 weeks	More robust improvements across BMI, FM, WC, %BF; some increases in MM/TBW.	Thomson et al., 2018^12^ (12 weeks online nutrition); Chiu et al., 2017^15^ (12 weeks aerobic); Flack et al., 2020^2^ (12 weeks exercise)
14-24 weeks	Comprehensive improvements in adiposity and lean mass outcomes.	Guerendiain et al., 2019^19^ (16 weeks Zumba); Hernandez-Reyes et al., 2019^13^ (24 weeks physical activity); Rosi et al., 2020^14^ (24 weeks Mediterranean diet)
>1 year	Sustained reductions in adiposity; improvements in lean mass retention, CV risk.	Abulmeaty et al., 2016^39^ (12 month multimodal life Style)
Regional anomaly	Paradoxical increases in BMI/FM despite structured programs.	Shandu et al., 2023^17^ (8 weeks HIIT, Africa)

**Effects of Intervention on Body Composition**

This review identified regional and temporal patterns in body composition outcomes, suggesting that geographical context influences intervention efficacy. Consistent with Sandercock & Andrade (2018), longer and supervised programs in North America and Europe tended to show greater improvements in FM, MM, and TBW, emphasizing the role of professional.

In Asia, innovative formats such as aerobic exercise and virtual Zumba reported meaningful reductions in FM and WC.^15,16^ These findings support the growing feasibility of hybrid or remote programs, particularly when sustained participant contact and motivational support are incorporated.^23^ In contrast, outcomes in Africa, such as those reported by Shandu et al. (2023), were less consistent, likely reflecting structural challenges including limited access to infrastructure, food insecurity, and cultural perceptions of body size.^17,25,28^ Community-based approaches adapted to local norms remain promising but require stronger implementation fidelity.^29^

Meta-analytic results indicated that TBW showed the most consistent and statistically significant improvements, with low heterogeneity (I2 = 0%). By contrast, changes in MM and FFM were small and not statistically significant across subgroups. These differences likely reflect physiological mechanisms: TBW responds rapidly to hydration status, dietary intake, and short-term exercise, whereas MM and FFM adapt more slowly, requiring prolonged resistance training and sustained nutritional support.^30,31^ The distinct biological processes—fluid balance versus tissue remodeling—explain why TBW is more sensitive to short-term interventions, while MM and FFM improvements become apparent only in longer or multimodal programs.^32,33^

Overall, these findings underscore the importance of aligning intervention goals with physiological responsiveness: short- to medium-term programs may be effective for TBW-related outcomes, while longer, resistance-focused interventions are required to achieve measurable gains in lean tissue.

### EFFECTIVENESS BASED ON TYPE OF INTERVENTION

**Exercise-Based Interventions**

Exercise-only interventions demonstrated variable efficacy across regions. Structured, longer-duration programs in Europe and Asia produced the most notable reductions in FM.^13,15,34^ By contrast, North American studies reported limited effects, likely due to shorter intervention duration and lower behavioral reinforcement.^2,7^

Overall, exercise alone did not produce consistent or statistically significant improvements in lean mass (MM or FFM), consistent with Menon et al. (2022).^5^ Notably, exercise type (aerobic vs resistance) was not analyzed separately in this review, which is an important limitation since lean mass outcomes are generally more responsive to resistance training. The absence of stratification may therefore explain some of the variability and non-significant effects observed, underscoring the need for modality-specific analyses in future research.

In Africa, despite the implementation of structured exercise, outcomes were often limited by socio-cultural and economic barriers, as illustrated by Shandu et al. (2023).^17^ These findings emphasize the importance of context-specific, community-supported strategies to improve adherence and intervention fidelity.^26^

**Nutrition-Based Interventions**

Nutrition-focused interventions uniformly improved BMI and FM, particularly when involving fractured plans like the Mediterranean diet or energy restriction.^14,21^ However, lean mass (MM) and fat-free mass (FFM) changes were generally minimal and not statistically significant. Ashtary-Larky et al. (2017) reported that diet-induced weight loss can reduce lean mass, a limitation consistently noted across studies, suggesting that nutrition alone may be insufficient to preserve muscle tissue.^21^

Digital formats improved accessibility and scalability but require strategic reinforcement to match the engagement levels of inperson counseling.^5,12^ These findings highlight the necessity of pairing dietary interventions with physical activity to preserve lean mass and promote metabolic health.

**Combined Interventions (Exercise + Nutrition)**

Combined interventions consistently delivered superior outcomes across multiple metrics. Studies by Gottesman et al. (2018), Sengün et al. (2024), and Quist et al. (2022) demonstrated synergistic improvements in BMI, FM, TBW, and modest changes in lean mass.^6,11,35^ This synergism arises from caloric restriction through diet and enhanced energy expenditure and muscle preservation via exercise.^3,22^

The meta-analysis confirmed these findings, showing significant improvements in BMI (SMD = -1.74; p = .01), %BF (SMD = -2.40; p < .00001), and TBW (SMD = 1.54; p < .00001). By contrast, muscle mass (SMD = 0.27; p = .60) did not increase significantly. These non-significant results highlight the challenge of achieving lean mass gains in combined programs and indicate that future studies should test higher-intensity exercise, resistance-based protocols, or longer follow-up periods to determine whether clinically meaningful increases in MM or FFM can be achieved.

The comprehensive nature of combined interventions aligns with WHO recommendations and restects behavioral reinforcement mechanisms, whereby participation in one activity (e.g., group exercise) supports adherence to another (e.g., healthy eating). Such integrated models also address gender-specific and sociocultural motivators, contributing to improved adherence and more favorable outcomes.^35,36^

## CONCLUSION

This systematic review confirms that the duration and type of lifestyle intervention significantly shape their effectiveness in altering body composition among adults. Longer-duration programs, especially those integrating both dietary and exercise components, tend to produce the most consistent and holistic improvements. While short-term interventions may initiate changes, their sustainability remains limited unless reinforced by structured support or higher intensity. The interplay between intervention length, cultural context, and adherence emerged as a crucial factor influencing outcomes, particularly in underrepresented regions.

Combined interventions consistently outperform exercise-only or diet-only approaches, especially in terms of fat mass reduction and total body water improvement. However, gains in muscle mass and lean tissue remain modest across most modalities, suggesting a need for refined strategies targeting resistance training or protein optimization. Notably, variations in effectiveness across geographic settings point to the importance of culturally tailored designs and locally relevant implementation strategies.
